# Differential Effects of Curcumin and Cordycepin on Oral Squamous Cell Carcinoma Cells: ROS-Mediated Cytotoxicity and Real-Time Morphological Analysis

**DOI:** 10.3390/molecules31071221

**Published:** 2026-04-07

**Authors:** Bianca Voicu Balasea, Miruna-Silvia Stan, Radu Radulescu, Ana Cernega, Kersti Alm, Monica Musteanu, Florentina Rus, Alexandra Ripszky, Silviu Mirel Pituru

**Affiliations:** 1 The Interdisciplinary Center for Dental Research and Development, “Carol Davila” University of Medicine and Pharmacy, 19–21 Jean Louis Calderon, 030167 Bucharest, Romania; bianca.voicu-balasea@drd.umfcd.ro (B.V.B.); silviu.pituru@umfcd.ro (S.M.P.); 2 Department of Biochemistry and Molecular Biology, Faculty of Biology, University of Bucharest, 91–95 Splaiul Independentei, 050095 Bucharest, Romania; miruna.stan@bio.unibuc.ro; 3 Department of Biochemistry, Faculty of Dental Medicine, “Carol Davila” University of Medicine and Pharmacy, 17–23 Plevnei Street, 020021 Bucharest, Romania; radu.radulescu@umfcd.ro; 4 Department of Organization, Professional Legislation and Management of the Dental Office, Faculty of Dental Medicine, “Carol Davila” University of Medicine and Pharmacy, 17–23 Plevnei Street, 020021 Bucharest, Romania; ana.cernega@umfcd.ro; 5 Phase Holographic Imaging AB, 223 70 Lund, Sweden; kersti.alm@phiab.com; 6 Department of Biochemistry and Molecular Biology, Faculty of Pharmacy, University Complutense of Madrid, 28040 Madrid, Spain; mmustean@ucm.es

**Keywords:** curcumin, cordycepin, oral squamous cell carcinoma, gingival epithelial cells, ROS, apoptosis, morphology

## Abstract

Oral squamous cell carcinoma (OSCC) remains a major clinical challenge, highlighting the need for novel therapeutic strategies. Natural bioactive compounds such as curcumin (Cu) and cordycepin (Co) have shown anticancer potential; however, their effects on cancer cell morphology and behavior remain incompletely characterized. This study assessed the individual and combined effects of Cu and Co on oral squamous cell carcinoma cells (OECM-1) and normal human gingival epithelial cells (HGEpiC) over 24 and 48 h. Metabolic activity, membrane integrity, oxidative stress, apoptosis, and inflammatory responses were evaluated using MTT, LDH, ROS-H_2_O_2_, caspase 3/7, and NO assays. Label-free digital holographic microscopy enabled real-time monitoring of morphology, motility, and proliferation. Both compounds induced ROS-mediated cytotoxicity, but responses were notably more pronounced in OECM-1 than in HGEpiC cells. Real-time morphological profiling revealed distinct response patterns: Co primarily exerted cytostatic effects, whereas Cu induced cell shrinkage, impaired motility, and inhibited cell division. The combination treatment (CC) largely reflected Cu-driven morphological and functional changes, with Co coexisting without counteracting Cu’s effects. Taken together, these findings reveal compound-specific mechanisms of action for Cu and Co in OSCC therapy.

## 1. Introduction

OSCC accounts for approximately 90% of oral malignancies [1], remaining a major global health challenge within head and neck oncology. It is characterized by an unfavorable prognosis and high mortality, with approximately 177,000 deaths reported annually [2]. According to the Global Cancer Observatory (GCO), an estimated 400,000 new cases of oral cancer were diagnosed worldwide in 2022 [3]. The high mortality and global burden of OSCC underscore the need for novel therapeutic strategies.

In this context, natural bioactive compounds with antitumor properties have attracted considerable attention in OSCC therapy. *Cordyceps* is a well-known medicinal fungus in traditional Chinese medicine and is often referred to as a “miracle mushroom” due to its remarkable health-promoting properties. It has a unique life cycle and predominantly grows on the larvae of a specific moth species, *Hepialus armoricanus* Oberthur (Lepidoptera). Among the various species, *Cordyceps militaris* produces high levels of cordycepin (Co). The chemical structure of Co closely resembles that of adenosine, differing only by the absence of a hydroxyl group at the 3′ position of the ribose moiety. This structural similarity to adenosine underlies its wide range of nutraceutical and therapeutic effects, including antitumor, anti-inflammatory, antimicrobial, anti-diabetic, hypolipidemic, analgesic, and immunomodulatory activities [4]. Current literature indicates that Co can induce autophagy to suppress FAK and Akt phosphorylation, as well as MMP2 and MMP9 activity, leading to reduced migration and invasion of HSC-4 oral cancer cells [5]. Additionally, Co has been shown to enhance radiosensitivity in OSCC by promoting autophagy and apoptosis through cell cycle arrest [6]. Curcumin (Cu) is the active polyphenol in turmeric (*Curcuma longa*). Turmeric is a rhizomatous, herbaceous perennial plant belonging to the ginger family, widely used both as a culinary spice and in medical and scientific research. The rhizome of this plant contains Cu (1,7-bis(4-hydroxy-3-methoxyphenyl)-1,6-heptadiene-3,5-dione), also known as diferuloylmethane, a natural polyphenol with well-documented antioxidant, anti-inflammatory, antimutagenic, antimicrobial, and anticancer properties [7]. Cu has been extensively studied in the context of cancer, including oral cancer. In OSCC, Cu has been reported to induce ROS-mediated apoptosis [8], decrease cell proliferation and viability [9], and trigger both apoptosis and autophagy [10]. Furthermore, it modulates molecular signaling pathways such as NF-κB, Wnt/β-catenin, and EGFR [11].

Although previous studies have evaluated the effects of Cu or Co on autophagy, apoptosis, and proliferation in OSCC, none have performed real-time monitoring of morphological changes in OECM-1 cells following treatment with either compound. Moreover, while prior research has described the Cu–Co (CC) combination and demonstrated its synergistic anticancer and antibacterial effects in vitro in HeLa cells [12] and SH-SY5Y cells [13], its effects have not yet been explored in HGEpiC or OECM-1 cells. This gap guided our choice of compounds.

In this study, normal HGEpiC and malignant OECM-1 cells were selected based on their shared gingival epithelial origin, allowing a direct comparison of physiological and malignant oral epithelial responses to treatment. According to the literature, localized administration may enhance therapeutic effects on malignant oral cells while potentially reducing systemic toxicity commonly associated with conventional treatments [14].

The novelty of our approach lies in the combined use of Cu and Co, coupled with real-time monitoring of OECM-1 cell morphology, motility, and proliferation, alongside measurements of ROS, apoptosis, and metabolic activity via MTT assays. Accordingly, our study aims to assess the potential of localized therapy by investigating the effects of Cu and Co, individually and in combination, on HGEpiC and OECM-1 cells.

## 2. Results

### 2.1. Differential Cytotoxicity of Co, Cu, and CC on HGEpiC and OECM-1 Cells

**Figure 1:**
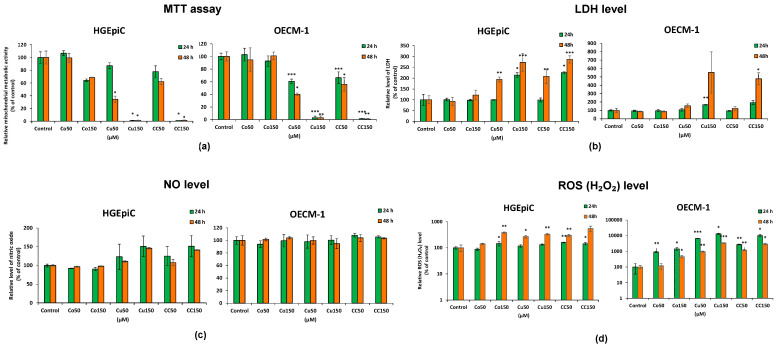
Comparative analysis of the effects of Co, Cu, and CC treatments (50 μM and 150 μM) on HGEpiC and OECM-1 cells after 24 and 48 h. (**a**) Quantification of the metabolic mitochondrial activity by MTT assay; (**b**) quantification of released LDH level; (**c**) quantification of released NO level; (**d**) quantification of ROS (H_2_O_2_) relative luminescence intensity. The *y*-axis is plotted on a log_10_ scale to allow visualization of large increases. Controls represent untreated cells. Data are presented as mean ± SD (*n* = 3). * *p* < 0.05; ** *p* < 0.01; *** *p* < 0.001 vs. control (one-way ANOVA followed by Tukey’s post hoc test; Welch’s ANOVA followed by the Games–Howell post hoc test; Kruskal–Wallis test followed by Dunn’s post hoc).

The results of the MTT assay (Figure 1a) showed that treatments with Co, Cu, and the CC combination reduced metabolic activity in both investigated cell lines, with the effect being concentration- and time-dependent. The most pronounced decreases were observed for Cu150 and CC150. In the case of HGEpiC cells, after 24 h, a statistically significant decrease in metabolic activity was observed only at high concentrations, namely Cu150 (−98%) and CC150 (−98%), compared to the control. After 48 h, metabolic activity was significantly reduced at Cu50 (−65%), Cu150 (−97%), and CC150 (−98%) compared to the control. In the OECM-1 cells, after 24 h of exposure, metabolic activity decreased statistically significantly compared to the control in Cu50 (−39%), CC50 (−34%), Cu150 (−97%), and CC150 (−98%). After 48 h, the reduction in metabolic activity was even more pronounced, being statistically significant in Cu50 (−60%), CC50 (−44%), Cu150 (−97%), and CC150 (−98%) compared to the control.

The IC_50_ data (Table 1) indicate that Co was essentially non-cytotoxic in both HGEpiC and OECM-1 cells, with values exceeding 150 µM at both 24 and 48 h. In contrast, Cu exhibited moderate, time-dependent cytotoxicity. The CC treatment shows slightly lower toxicity than Cu alone. Similar IC_50_ values in HGEpiC and OECM-1 cells indicate no cell selectivity, suggesting that Cu drives the effect without synergism from Co.

The results of the LDH assay (Figure 1b) indicated an increase in LDH release, especially under Cu exposure conditions, suggesting impairment of cell membrane integrity. In the HGEpiC cells, after 24 h, a significant increase in LDH levels was observed only at Cu150 (+113%) and CC150 (+125%) compared to the control. After 48 h, LDH levels increased significantly at Cu50 (+93%), CC50 (+108%), Cu150 (+172%), and CC150 (+186%) compared to the control. In the OECM-1 cells, after 24 h, an increase in LDH levels of (+68%) was observed at Cu150. After 48 h, very large increases in LDH were recorded at CC150 (+376%, *p* < 0.05) compared to the control. Although LDH levels increased by 452% at Cu150 compared to the control, this change was not statistically significant (*p* > 0.05). These results are consistent with the data obtained in the MTT assay.

Regarding nitric oxide (Figure 1c), in HGEpiC cells, a statistically insignificant increase in NO levels compared to the control was observed after 24 h and 48 h of exposure to Cu150 and CC150. In the case of the OECM-1 cells, no significant differences in NO levels were observed compared to the control.

ROS levels (Figure 1d) were increased in both cell lines, with the effect being much more pronounced in OECM-1. In HGEpiC cells, after 24 h, increases in ROS were recorded for Co150 (+43%), CC50 (+59%), and CC150 (+43%), while after 48 h, increases were observed for Co150 (+284%), Cu50 (+170%), CC50 (+196%), and Cu150 (+234%). In the OECM-1 cells, after 24 h, ROS levels increased significantly for Co50 (+808%), Co150 (+1346%), Cu50 (+6469%), Cu150 (+13,118%), CC50 (+2606%), and CC150 (+10,030%). After 48 h, increases in ROS were observed for Co150 (+355%), Cu50 (+860%), CC50 (+1102%), Cu150 (+3309%), and CC150 (+2858%).

### 2.2. Caspase-3/7 Activity After Exposure of HGEpiC and OECM-1 Cells to Co, Cu, and CC

**Figure 2:**
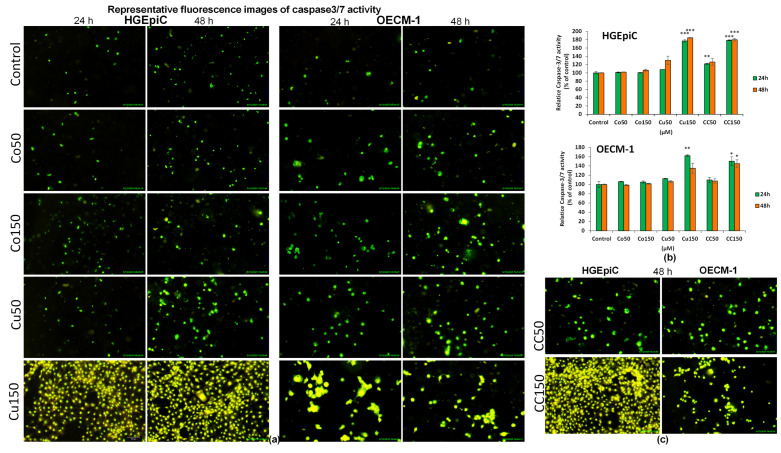
Late apoptosis after Co, Cu, and CC treatments (50 μM and 150 μM) on HGEpiC and OECM-1 cells after 24 and 48 h. (**a**) Representative fluorescence microscopy images of caspase-3/7 activity in HGEpiC and OECM-1 cells. Scale bar: 100 µm (applies to all images); (**b**) quantification of relative fluorescence intensity of caspase-3/7 activity using FLUOstar Omega microplate reader; (**c**) representative fluorescence microscopy images of caspase-3/7 activity in HGEpiC and OECM-1 cells after 48 h exposure to CC50 and CC150. Scale bar: 100 µm (applies to all images). Panels (**a**) and (**c**): treated cells compared to the common control shown in (**a**). Controls represent untreated cells. Data are presented as mean ± SD (*n* = 3). * *p* < 0.05; ** *p* < 0.01; *** *p* < 0.001 vs. control (Welch’s ANOVA followed by the Games–Howell post hoc test).

Fluorescence images (Figure 2a) showed an increase in fluorescence intensity, directly proportional to the activity of caspase-3/7, the executioner protein of apoptosis, compared to the control, both following Co and Cu treatment, in HGEpiC and OECM-1 cells.

In HGEpiC cells, fluorescence intensity (Figure 2b) increased statistically significantly after 24 h in the case of CC50 (+21%), Cu150 (+76%), and CC150 (+78%), and after 48 h for Cu150 (+84%) and CC150 (+80%). In OECM-1 cells, fluorescence intensity (Figure 2b) increased significantly after 24 h for Cu150 (+62%) and CC150 (+50%), and after 48 h for CC150 (+45%).

In Figure 2c, CC50 and CC150 at 48 h in both HGEpiC and OECM-1 cells showed a higher caspase-3/7 fluorescence intensity compared to the control. These results were similar to those observed after exposure to Cu50 and Cu150. The highest activation of caspase-3/7 was observed in the case of Cu150 and CC150 treatment for both cell lines.

### 2.3. Digital Holographic Microscopy Shows That Co-Induced Apoptotic Cell Death Does Not Completely Inhibit Cell Division

**Figure 3:**
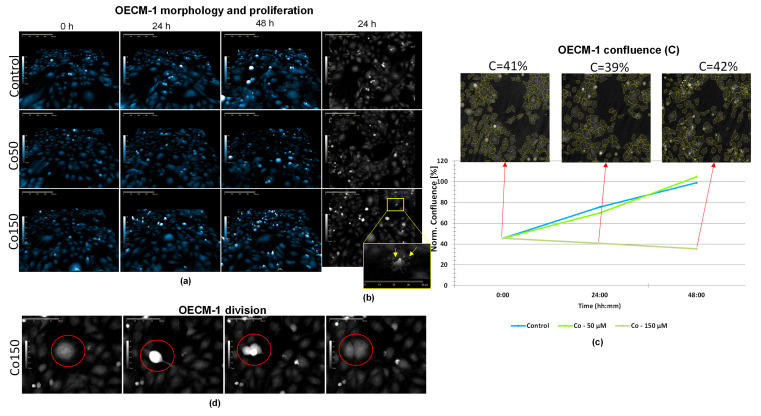
HoloMonitor^®^ M4 phase images and analysis of OECM-1 cells after Co treatment (50 and 150 µM). (**a**) Representative holographic phase 3D images of OECM-1 cells after exposure to Co50 and Co150 for 0, 24 and 48 h; (**b**) representative holographic phase 2D images of OECM-1 cells after exposure to Co50 and Co150 for 24 h, showing apoptotic morphology, with apoptotic bodies clearly indicated (yellow arrow); (**c**) OECM-1 confluence (C) at 0, 24 and 48 h; images represent Co150-treated cells. Data are presented as mean ± SD (*n* = 3). ** *p* < 0.01, Co150 vs. Co50 (48 h); * *p* < 0.05, Co150 vs. control (48 h); * *p* < 0.05, 0 h vs. 24 h (Co50); *** *p* < 0.001, 0 h vs. 48 h (Co50); *** *p* < 0.001, 24 h vs. 48 h (Co50) (one-way ANOVA followed by Tukey’s post hoc test). (**d**) OECM-1 cell division after treatment with Co150.

HoloMonitor^®^ M4 phase images (Figure 3a) showed that OECM-1 cells exposed to Co treatment (50 and 150 µM), as well as control cells, continued to proliferate over time. Even after 48 h of Co150 treatment, thick and rounded-up cells were observed, representing a morphology specific to cell division. Analysis of cell confluence (Figure 3c) indicated a progressive increase in cell density over time in both the control and Co50 groups (*p* < 0.05, 0 h vs. 24 h; *p* < 0.001, 0 h vs. 48 h; *p* < 0.001, 24 h vs. 48 h, one-way ANOVA followed by Tukey’s post hoc test). In contrast, the confluence of OECM-1 cells exposed to Co150 remained approximately constant (~C = 40%) throughout the observation period, being significantly lower than that of Co50 (*p* < 0.01) and control (*p* < 0.05) after 48 h. Therefore, although OECM-1 cell density did not increase at Co150 (Figure 3c), cell division was still present (Figure 3d), and at the same time, there were also cells showing apoptotic morphology with clearly seen apoptotic bodies (Figure 3b).

### 2.4. Digital Holographic Microscopy Shows Cu-Specific Effects on Cell Volume, Morphology, and Structural Integrity

**Figure 4:**
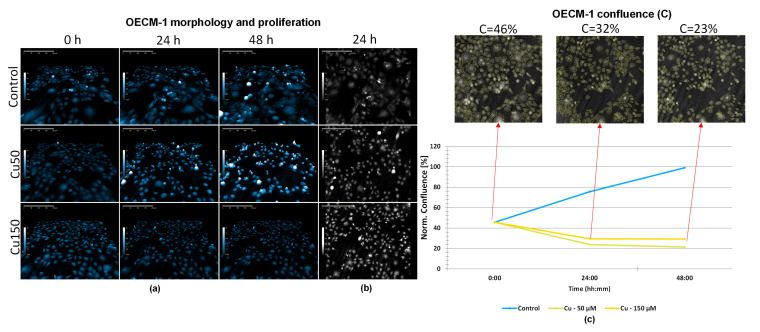
HoloMonitor^®^ M4 phase images and analysis of OECM-1 cells after Cu treatment (50 and 150 µM). (**a**) Representative holographic phase 3D images of OECM-1 cells after 0, 24, and 48 h of exposure to Cu50 or Cu150 μM; (**b**) representative holographic phase 2D images of OECM-1 cells after 24 h after exposure to Cu50 and Cu150; (**c**) OECM-1 confluence at 0, 24, and 48 h; images represent Cu150-treated cells. Data are presented as mean ± SD (*n* = 3). *** *p* < 0.001, 0 h vs. 24 h (Cu50); *** *p* < 0.001, 0 h vs. 48 h (Cu50); *** *p* < 0.001, 0 h vs. 24 h (Cu150); *** *p* < 0.001, 0 h vs. 48 h (Cu150) (one-way ANOVA followed by Tukey’s post hoc test).

The images (Figure 4a) suggested that OECM-1 cells exposed to Cu50 appeared less confluent over 24 and 48 h compared to time 0, which may reflect smaller cell size and reduced proliferation. A similar trend was observed in the case of exposure to Cu150; the three-dimensional cellular structure progressively diminished over time, and the cellular density remained similar to that at time 0. These observations were confirmed by 2D images (Figure 4b). Analysis of the surface area occupied by the cells (Figure 4c) revealed a decrease over time for Cu150 from 43% at time 0 to 32% after 24 h, and only 23% after 48 h, indicating cell shrinkage. This reduction was statistically significant for 0 h vs. 24 h (*p* < 0.001) and 0 h vs. 48 h (*p* < 0.001) as determined by one-way ANOVA followed by Tukey’s post hoc test. Similarly, Cu50-treated cells showed a significant decrease in confluence over time, from baseline to 24 h (*p* < 0.001) and from baseline to 48 h (*p* < 0.001). There was no indication of cell division taking place in the Cu-treated cells.

### 2.5. Digital Holographic Microscopy Shows That CC Treatment Enhances Antitumoral Effects and Inhibits Proliferation and Motility

**Figure 5:**
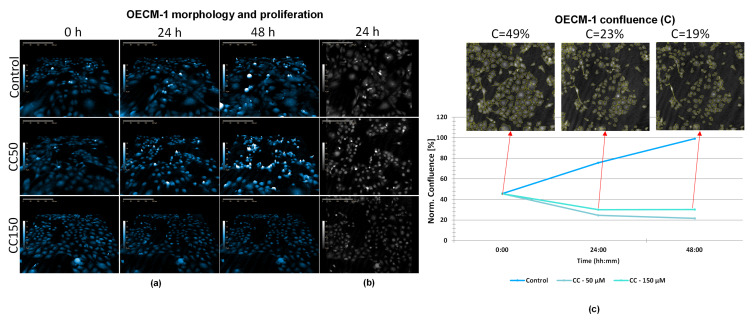
HoloMonitor^®^ M4 phase images and analysis of OECM-1 cells after CC treatment (50 and 150 µM). (**a**) Representative holographic phase 3D images of OECM-1 cells after 0, 24, and 48 h of exposure to CC50 or CC150; (**b**) representative holographic phase 2D images of OECM-1 cells after 24 h after exposure to CC50 and CC150; (**c**) OECM-1 confluence at 0, 24, and 48 h; images represent CC150-treated cells. Data are presented as mean ± SD (*n* = 3). * *p* < 0.05, 0 h vs. 24 h (CC150); * *p* < 0.05, 0 h vs. 48 h (CC150) (one-way ANOVA followed by Tukey’s post hoc test).

Following exposure of OECM-1 cells to the combination of CC50 and CC150 (Figure 5a), it was observed that OECM-1 cells reacted morphologically and behaviorally like cells exposed to Cu50 or Cu150, respectively. Control cells displayed a larger, well-spread morphology, whereas CC50-treated cells were clearly smaller, and CC150-treated cells appeared very small. Cell division and motility were completely inhibited after exposure to CC150. These observations were confirmed by 2D images (Figure 5b). Confluence analysis indicated a progressive reduction in the surface area occupied by cells over time, decreasing from 49% at baseline to 23% after 24 h and 19% after 48 h (Figure 5c). This decrease was statistically significant for 0 h vs. 24 h (*p* < 0.05) and 0 h vs. 48 h (* *p* < 0.05) as determined by one-way ANOVA followed by Tukey’s post hoc test.

### 2.6. Single-Cell Tracking Shows That Cu Induces Progressive Cell Shrinkage in OECM-1 Cells

**Figure 6:**
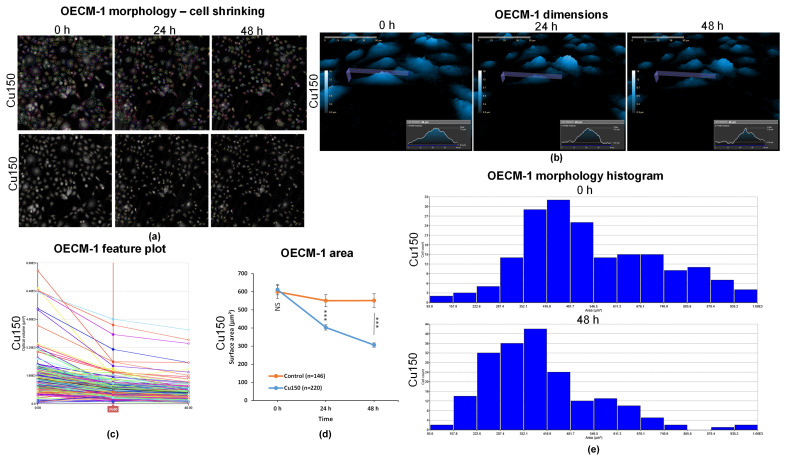
Morphological analysis of individual OECM-1 cells over time after Cu150 treatment. (**a**) Representative holographic phase images showing the tracking of OECM-1 cells at 0, 24, and 48 h after exposure to Cu150; (**b**) representative holographic phase 3D images illustrating OECM-1 cell dimensions at 0, 24, and 48 h after exposure to Cu150; (**c**) optical volume (µm^3^) of OECM-1 cells after 0, 24, and 48 h of exposure to Cu150; (**d**) surface area (µm^2^) of OECM-1 cells after 0, 24, and 48 h of exposure to Cu150 compared with control (unexposed) cells. Data are presented as mean ± SD (control *n* = 146; Cu150 *n* = 220); *** *p* < 0.001 vs. control (Mann–Whitney U test); NS, non-significant. (**e**) Histogram of OECM-1 cell area (µm^2^) at 0 and 48 h after exposure to Cu150 (0 h, *n* = 186; 48 h, *n* = 195). *** *p* < 0.001 Mann–Whitney U test.

For a more detailed understanding of the morphological changes induced by Cu150 treatment, in Figure 6a, cells were individually marked and tracked over time. Individual tracking showed progressive cell shrinkage (Figure 6a), with decreases in height and length after 24 and 48 h (Figure 6b).

The distribution of the cell population according to optical volume (µm^3^) in Figure 6c showed a decrease after 24 h, which remained reduced or decreased even further after 48 h. Figure 6d shows a progressive reduction in cell area in Cu150-treated OECM-1 cells at 24 and 48 h, whereas control cells maintained their size (mean ± SD: Control, 0 h = 599.5 µm^2^, 24 h = 551.5 µm^2^, 48 h = 549.5 µm^2^; Cu150, 0 h = 611.7 µm^2^, 24 h = 403.1 µm^2^, 48 h = 306.3 µm^2^). The mean area of Cu150-treated cells decreased by 34% at 24 h and 50% at 48 h compared with baseline. The Mann–Whitney U test confirmed that cell areas in Cu150-treated cells were significantly smaller than in control at both 24 h and 48 h (*p* < 0.001).

Figure 6e provides a more detailed view of the cell population distribution (only cells with areas between 93 and 1000 µm^2^ were considered for this analysis). At 0 h, most OECM-1 cells exposed to Cu150 had areas between 352 and 546 µm^2^. Notably, at 0 h, a considerable number of cells had larger areas (546–1000 µm^2^), whereas after 48 h, these larger cells were rare or absent. After 48 h, the majority of cells had areas between 222 and 481 µm^2^, indicating pronounced cell shrinkage. The mean area decreased from 541.7 µm^2^ at 0 h to 396.0 µm^2^ at 48 h, representing a 27% reduction. The Mann–Whitney U test confirmed that this decrease was highly significant (*p* < 0.001).

### 2.7. Cu Treatment Inhibits Cell Motility and Migration of OECM-1 Cells

**Figure 7:**
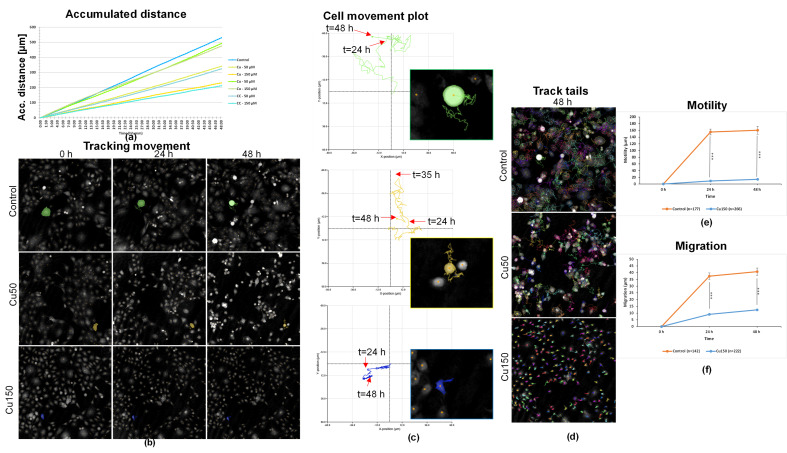
HoloMonitor^®^ M4 phase images and analysis of OECM-1 cells after Cu treatment (50 and 150 µM). (**a**) Accumulated distance (µm) of OECM-1 cells after 48 h of exposure to Cu50, Cu150, Co50, Co150, CC50, and CC150 compared with control (unexposed) cells; (**b**) representative holographic phase images showing the tracking of OECM-1 cell movement at 0, 24, and 48 h after exposure to Cu50 and Cu150 compared with control (unexposed) cells; (**c**) cell movement plots illustrating the tracking of OECM-1 cell movement at 0, 24, and 48 h after exposure to Cu50 and Cu150 compared with control (unexposed) cells (control, green; Cu50, yellow; Cu150, red); (**d**) track tails of OECM-1 cells after 48 h of exposure to Cu50 and Cu150 compared with control cells; (**e**) motility (µm) of OECM-1 cells at 0, 24, and 48 h after exposure to Cu150 compared with control (unexposed) cells. Data are presented as mean ± SD (control *n* = 177; Cu150 *n* = 266); *** *p* < 0.001 vs. control (Mann–Whitney U test). (**f**) Migration (µm) of OECM-1 cells at 0, 24, and 48 h after exposure to Cu150 compared with control (unexposed) cells. Data are presented as mean ± SD (control *n* = 142; Cu150 *n* = 222); *** *p* < 0.001 vs. control (Mann–Whitney U test).

Analyzing OECM-1 cell motility through the accumulated distance of the cell populations over 48 h indicates that treatment with Co50 or 150 did not significantly affect motility, while treatment with Cu50 or Cu150, as well as with CC50 and CC150, had a major impact on cell motility (Figure 7a).

To highlight the motility and migration following treatment with Cu50 and Cu150, a representative cell for each experimental condition was individually marked and tracked in Figure 7b. The cell movement plots (Figure 7c), together with Figure 7b, showed that the representative cell in the control group (green) moved large distances both after 24 h and 48 h. Also, the representative cell for Cu50 (yellow) showed motility over time. In contrast, in the case of Cu150, the very small observed movement of the representative cell (red) did not reflect real displacement in the visual field or in the well but was the result of the cellular shrinkage process.

Figure 7d, showing the tracking of the movements of the cell population at 30 min intervals, indicated high motility in the control, moderate motility in Cu50, and absent motility in Cu150. The motility (Figure 7e) and migration (Figure 7f) plots highlighted clear differences between control and Cu150-treated cells. Motility and migration of Cu150-treated cells were significantly lower than control at both 24 h and 48 h (Mann–Whitney U test, *p* < 0.001). The detected movements of Cu150-treated cells corresponded primarily to cellular shrinkage, confirming immobilization of the cells after treatment.

In conclusion, the biochemical analysis revealed differences between HGEpiC and OECM-1 cells. In HGEpiC, treatments with Co, Cu, and the CC combination caused significant decreases in metabolic activity, along with increases in LDH release and ROS production, particularly at 48 h. In OECM-1 cells, the same treatments induced more pronounced reductions in metabolic activity and increases in LDH and ROS. Data obtained using digital holographic microscopy in OECM-1 cells further supported these findings. In the case of Co, OECM-1 cells exposed to Co150 maintained an approximately constant confluence (~40%) throughout 48 h, accompanied by elevated ROS levels. For Cu and the CC combination, morphological alterations, including reduced cell volume and inhibition of cell division and migration, were accompanied by decreased mitochondrial metabolic activity, increased ROS production, and caspase-3/7 activation. Together, these findings suggest that treatment-induced biochemical changes were paralleled by alterations in cellular morphology and behavior.

## 3. Discussion

This study evaluated the effects of Cu, Co, and their combination CC on HGEpiC and OECM-1 cells in order to provide a basis for exploring their potential as localized anti-cancer therapies in OSCC.

The results of the MTT assay revealed a concentration- and time-dependent reduction in mitochondrial metabolic activity in both cell lines, with the most pronounced effects observed following treatments with Cu150 and CC150. Notably, OECM-1 cells exhibited a marked reduction in metabolic activity as early as 24 h at lower concentrations (Cu50), whereas HGEpiC cells were significantly affected only after longer exposure times (48 h). In contrast, treatment with Cu150, alone or in combination (CC150), induced a pronounced and comparable reduction in metabolic activity in both cell lines, leading to an almost complete loss of mitochondrial function. These findings indicate a high sensitivity of OECM-1 cells at lower concentrations and earlier time points, while high-dose Cu exerts a strong cytotoxic effect irrespective of cell type. This observation is in partial agreement with previous reports describing selective antitumor activity of Cu or its derivatives without significant effects on normal cells [15]. Consistent with these observations, LDH levels increased following CC150 treatments in both HGEpiC and OECM-1 cells, with a more pronounced loss of membrane integrity observed in OECM-1 cells, suggesting a potential selective vulnerability of malignant cells to high-dose Cu and CC treatments. LDH release is considered a reliable biomarker of cytotoxicity [16], and it is associated with necrotic cell death, as LDH leakage occurs at higher levels during necrosis than during apoptosis, as reported by Akira Sato et al. (2023); however, moderate LDH release is also consistent with late-stage apoptosis [17]. In our study, the concomitant increase in LDH and caspase-3/7 activity suggests that Cu150 and CC150 may induce mixed cell death mechanisms in OECM-1 cells, involving both necrosis and apoptosis. This dual mechanism is consistent with previous reports of Cu-induced apoptosis and necrosis in oral cell lines, leading to DNA damage and inhibition of cancer progression [14]. Similarly, increased LDH release following Cu treatment has been observed in both sensitive (MCF7, HCT116, A549) and resistant (MCF7/TH, HCT116R, A549/ADR) cancer cell lines, further supporting its cytotoxic efficacy [16]. These findings highlight the cytotoxic effects of Cu150 and CC150 in OECM-1 cells, as reflected by decreased MTT activity, increased LDH release, and elevated caspase-3/7 activity, supporting their potential as effective strategies for targeting OSCC.

Physiologically, NO levels are generally higher in OSCC than in normal tissue, where NO contributes to tumor progression [18]. Cu has been reported to exert anti-inflammatory effects, in part by reducing nitric oxide levels through downregulation of iNOS and modulation of NF-κB signaling [19,20]. In the present study, NO levels remained unchanged in OECM-1 cells and showed only a non-significant increase in HGEpiC cells. These findings indicate that Co and Cu treatments did not induce either a pro- or anti-inflammatory response in the tested cells, which may be advantageous for potential local therapeutic applications in the oral cavity.

Reactive oxygen species (ROS, measured as H_2_O_2_) levels increased following treatment with Co, Cu, and CC. While ROS levels increased only slightly over time in HGEpiC cells, a markedly stronger response was observed in OECM-1 cells, with higher ROS levels at 24 h than at 48 h, suggesting increased redox vulnerability at early time points and highlighting oxidative stress as a key mediator of cytotoxicity. The treatments appear to induce an initial oxidative stress, followed by either activation of antioxidant defenses or elimination of susceptible cells via apoptosis. Notably, the accumulation of ROS has been implicated in the modulation of regulated cell death pathways, including apoptosis, where elevated oxidative signals can promote apoptotic signaling and caspase activation [21]. More broadly, oxidative stress is recognized as a central determinant of cell fate and cell death signaling across multiple pathological contexts [22]. Consistent with this mechanism, increased ROS levels were accompanied by significant caspase-3/7 activation, suggesting that ROS play a central role in apoptosis induction in both HGEpiC and OECM-1 cells after Cu and CC treatments. These findings align with reports showing Cu-induced apoptosis mediated by ROS generation and reduced tumor formation in OSCC models [8], including effects of Cu analogs such as HO-3867 [23] and trienone 11 [24]. Similar ROS-dependent apoptotic effects in OSCC have been described for semilicoisoflavone B, naringenin, quercetin [25,26,27], and Co in various cancers, including cervical, bladder, and breast cancer [28,29,30]. Therefore, the differential ROS response observed between cell lines, with higher ROS accumulation in OECM-1 and a comparatively lower increase in HGEpiC, may indicate a potential therapeutic window. This suggests that Cu, Co, and CC treatments may preferentially exploit the redox vulnerability of OSCC cells, thereby promoting tumor cell damage.

Digital holographic microscopy enabled real-time, label-free monitoring of OECM-1 cells, providing insight into treatment-induced cellular responses. Although cell division was occasionally observed under Co150 treatment in OECM-1 cells, overall cell density remained constant, suggesting a cytostatic effect. Interestingly, cells displayed morphological features characteristic of classical apoptosis, including cell rounding, membrane blebbing, and formation of apoptotic bodies. However, in contrast to previous reports demonstrating Co-induced caspase-dependent apoptosis in OSCC [31], Co treatment alone did not result in a statistically significant increase in caspase-3/7 activity in our study. These findings suggest that, under our experimental conditions, Co primarily exerts cytostatic rather than cytotoxic effects, as cell density remained largely unchanged. This effect is consistent with previously reported mechanisms in other cancer models, where Co inhibits proliferation partly through MYC suppression, a key regulator of cell cycle progression [32].

In contrast, Cu150 and CC150 treatments exhibited a distinct morphological response, characterized by progressive OECM-1 cell shrinkage and reduction in optical volume, height, and surface area, suggesting profound cytoskeletal disruption without typical apoptotic bodies. This observation aligns with previous studies demonstrating that Cu disrupts microtubule stability, interferes with kinetochore–microtubule attachment, and perturbs mitotic spindle formation, leading to mitotic arrest and p53-dependent apoptosis [33]. Cu has also been shown to bind tubulin, impair microtubule assembly dynamics, and block cell cycle progression in HeLa and MCF-7 cells [34], potentially culminating in mitotic catastrophe [35]. In addition, Cu-induced actin cytoskeleton disorganization has been reported in A549 lung cancer cells, facilitating apoptotic signaling [36]. In our study, cell tracking analysis revealed high motility in control cells, moderate motility under low-dose Cu exposure, and complete immobilization following treatment with Cu150 or CC150. The observed movements were mainly due to cell shrinkage rather than actual motility, indicating reduced migratory behavior along with inhibition of cell division and thus proliferation, which may contribute to impaired cellular functionality of OECM-1 cells. This observation is relevant for OSCC, where cell motility plays an important role in local invasion and disease progression and may influence patient prognosis. In line with our findings, both in vitro and in vivo studies have shown that Cu inhibits oral cancer progression through suppression of proliferation, invasion, migration, angiogenesis, and induction of apoptosis and autophagy [37]. In OSCC models, Cu inhibited SCC-25 cell proliferation and invasion by suppressing EGFR signaling [11], reduced migration and invasion via downregulation of MMP10 [38], modulated MMP-2, MMP-9, EMT regulators, and p53 expression [39], and inhibited HGF-induced motility through c-Met blockade [40]. Moreover, Cu reduced OSCC migration and invasion in co-culture models by preventing the transformation of normal periodontal ligament fibroblasts into cancer-associated fibroblasts, thereby decreasing tumor aggressiveness and the reprogramming of surrounding normal cells [41]. Consequently, local Cu treatment could protect normal cells from being co-opted by tumor cells to support cancer progression.

Combination treatments with Cu and Co caused responses largely dominated by Cu, characterized by early and sustained inhibition of proliferation and motility, as well as morphology changes closely resembling the cellular phenotype induced by Cu alone. Consistent with this observation, MTT and LDH assays showed comparable effects between CC50 and Cu50, as well as between CC150 and Cu150. Accumulated distance analysis further confirmed that CC150 and Cu150 treatments resulted in the strongest inhibition of cell motility, followed by CC50 and Cu50 treatments, whereas the effects of Co50 and Co150 treatments remained close to control values. These findings indicate that Co does not counteract Cu-induced cell shrinkage or loss of motility but rather acts alongside Cu, which remains the primary driver of morphological changes, functional immobilization, and reduced proliferation in OECM-1 cells.

Taken together, the present findings suggest that Cu affects OECM-1 cell morphology, behavior, and redox balance, thereby impairing proliferative and migratory capacity, as reflected by reduced cell volume, motility, and metabolic activity. While Co150 alone induced only cytostatic effects and ROS increase in OECM-1 cells, the morphological and functional changes observed under Cu150 treatment were much more pronounced. In the combination treatment (CC), Co did not counteract the effects of Cu and may contribute mainly by sustaining ROS accumulation, while cell shrinkage and apoptotic response were predominantly driven by Cu. Such coordinated modulation of metabolic activity, oxidative stress, cell morphology, and apoptosis is particularly relevant in OSCC, where tumor progression depends on tightly regulated proliferation and invasive behavior.

## 4. Materials and Methods

### 4.1. Cell Culture

Two cell lines were used: HGEpiC cells (Innoprot, Elexalde Derio, Bizkaia, Spain; Cat. No. P10864) and OECM-1 cells (Sigma-Aldrich, St. Louis, MO, USA; Cat. No. SCC180).

HGEpiC cells were cultured in Epithelial Cell Medium-Plus (Innoprot, Elexalde Derio, Bizkaia, Spain; Cat. No. P60106-PLUS) supplemented with 5% fetal bovine serum (FBS), 1% EpiCGS (Epithelial Cell Growth Supplement, containing ITS, EGF, hydrocortisone, FGF-2), and 1% penicillin/streptomycin solution and maintained at 37 °C in a humidified atmosphere with 5% CO_2_. Culture flasks and 96-well plates were pre-coated with poly-L-lysine to enhance adhesion. Cells were seeded at 4 × 10^4^ cells/well in 96-well plates and incubated overnight prior to experiments.

OECM-1 cells were cultured in RPMI-1640 medium supplemented with 5% fetal bovine serum and 1% antibiotics at 37 °C and 5% CO_2_ [42]. Plates were not pre-treated with poly-L-lysine. Cells were seeded at the same density as HGEpiC cells (4 × 10^4^ cells/well) and incubated overnight before experiments.

### 4.2. Biocompounds

The biocompounds Co, a nucleoside analog extracted from *Cordyceps militaris* (≥98% purity, CAS: 73-03-0, Cayman Chemical, Ann Arbor, MI, USA), and Cu, a polyphenol extracted from turmeric (≥90% purity, CAS: 458-37-7, Cayman Chemical, Ann Arbor, MI, USA), were used in this study.

Compounds were dissolved in DMSO and sterilized by 1 h UV exposure. Before use, the stocks were diluted in the appropriate complete culture medium for each cell type to achieve final concentrations of 150 µM and 50 µM. For combination treatment, Cu and Co were mixed to achieve final concentrations of 50 µM + 50 µM or 150 µM + 150 µM (each compound at the indicated concentration). The final DMSO concentration was 0.5% in all experimental conditions, including the control group. The selected concentrations were based on preliminary viability testing and previously published studies [12].

### 4.3. Mitochondrial Metabolic Activity

Mitochondrial activity was assessed using the MTT assay (Biotium, Fremont, CA, USA; Cat No. 30006) after 24 and 48 h of treatment. MTT reagent was added per well at a 1:10 ratio with the culture medium, formazan crystals being solubilized with isopropanol after 4 h. Absorbance was read at 570 and 630 nm using a FLUOstar Omega microplate reader (BMG Labtech, Ortenberg, Germany). The biocompound concentration that produces 50% cell growth inhibition (IC50) was obtained by using the Quest Graph™ IC50 calculator (AAT Bioquest, Inc., Pleasanton, CA, USA) [43].

### 4.4. Lactate Dehydrogenase (LDH) Assay

Extracellular lactate dehydrogenase (LDH) release was used as an indicator of cell membrane integrity and, consequently, cytotoxicity. The assay was performed using the LDH Cytotoxicity Assay Kit (Merck, Darmstadt, Germany; Cat. No MAK529A). Briefly, 50 µL of culture medium was collected from each well to assess released LDH, and 80 µL of LDH reagent was added. Plates were incubated for 10 min at room temperature (approximately 20–25 °C) in the dark. Enzymatic activity was measured by reading absorbance at 500 nm using a FLUOstar Omega microplate reader (BMG Labtech).

### 4.5. Griess Assay

Extracellular nitric oxide (NO) production was evaluated using the Nitric Oxide Assay Kit (Merck, Darmstadt, Germany; Cat. No MAK454). The kit detects nitrites (NO_2_^−^), the main oxidation products of NO in aqueous solutions, via the Griess reaction. Culture medium was mixed with the Griess reagent at a 1:2 ratio (medium:reagent) in a 96-well plate. Absorbance was measured at 540 nm using a FLUOstar Omega microplate reader (BMG Labtech) after 1 h of incubation at 37 °C.

### 4.6. Reactive Oxygen Species (ROS)

Intracellular hydrogen peroxide (H_2_O_2_), a key reactive oxygen species, was measured using the ROS-Glo™ H_2_O_2_ Assay Kit (Promega, Madison, WI, USA; Cat. No. G8820) in HGEpiC and OECM-1 cells following treatment. The ROS-Glo™ reagent was added to the culture medium and incubated for 6 h at 37 °C. Luminescence was measured using the MyGlo^®^ Reagent Reader (Promega, Madison, WI, USA), with signal intensity reflecting the H_2_O_2_ levels in the samples.

### 4.7. Caspase-3/7 Activity (Fluorescence)

Caspase-3/7 activity was assessed in HGEpiC and OECM-1 cells after 24 and 48 h of treatment, with untreated cells serving as controls, using the BioTracker NucView 488 Green Caspase-3 Dye (Merck, Darmstadt, Germany; Cat. No. SCT100) to evaluate late apoptosis. The culture medium was replaced with PBS containing 5 µM of the dye, and cells were incubated for 30 min at room temperature in the dark. Fluorescence was imaged using an IM-3LD4D fluorescence microscope (Optika, Ponteranica, Italy) with excitation/emission wavelengths of 488/530 nm, and fluorescence intensity was quantified using a FLUOstar Omega microplate reader.

### 4.8. Live-Cell Imaging- HoloMonitor^®^ M4

Morphological changes, cell proliferation, and migration of OECM-1 cells were monitored using the HoloMonitor^®^ M4 digital holographic microscope (Phase Holographic Imaging PHI AB, Lund, Sweden). Cells were seeded in 96-well plates and exposed to Co, Cu, and the CC combination, with unexposed cells used as controls. A HoloLid™ (PHI AB) was placed on each plate to improve image quality and minimize evaporation. Time-lapse images were captured every 30 min for 48 h under standard culture conditions (37 °C, 5% CO_2_). Image analysis was performed using the HoloMonitor^®^ software (version 4.0.1.546; PHI AB, Göteborg, Sweden) to quantify cell confluence, area, optical volume, height, motility, and accumulated distance. Selected individual cells were manually tracked to assess morphological changes, including cell shrinkage, rounding, and motility over time.

### 4.9. Statistical Analysis

All statistical analyses were performed on raw data. For normally distributed variables, group comparisons were performed using one-way ANOVA or Welch’s ANOVA, along with Tukey HSD or Games-Howell post hoc tests. Non-parametric variables were compared using the Kruskal–Wallis H test with Dunn’s post hoc test. Results are presented as mean ± SD (*n* = 3). Comparisons between two independent groups for HoloMonitor^®^ M4 data were performed using the Mann–Whitney U test. The quantified data were converted to percent control (untreated cells) and plotted using Microsoft Excel. Statistical significance was considered when *p*-values were <0.05.

## 5. Conclusions

In conclusion, in HGEpiC, biochemical determinations (ROS) indicated comparatively milder responses to both Cu and Co, consistent with lower ROS levels than in OECM-1 and supporting the redox vulnerability of OECM-1 cells. Regarding OECM-1 cells, Cu affected cell morphology, inducing cell shrinkage, loss of motility, and apoptotic signaling, whereas Co exerted mainly cytostatic effects and contributed to ROS accumulation. The combination treatment (CC) reflected Cu-driven morphological and functional changes, with Co coexisting without counteracting its effects. Our results suggest that Cu and Co may act through complementary mechanisms and support further exploration for finding the best localized therapies in OSCC.

## Figures and Tables

**Table 1:** IC50 values (μg/mL) obtained by MTT assay for Co, Cu and CC treatments after 24 and 48 h of incubation with HGEpiC and OECM-1 cells. Data are expressed as means of three determinations.

	HGEpiC Cells		OECM-1 Cells	
	24 h	48 h	24 h	48 h
**Co**	>150 µM	>150 µM	>150 µM	>150 µM
**Cu**	70 µM	42 µM	57 µM	43 µM
**CC**	64 µM	56 µM	58 µM	53 µM

## Data Availability

The original contributions presented in the study are included in the article/Supplementary Material; further inquiries can be directed to the corresponding author.

## Supplementary Materials

The following supporting information can be downloaded at: https://www.mdpi.com/article/10.3390/molecules31071221/s1.

## Abbreviations

The following abbreviations are used in this manuscript:

Akt	Protein kinase B
ANOVA	Analysis of Variance
CC	Curcumin–Cordycepin combination
CC50	Curcumin–Cordycepin combination 50 µM
CC150	Curcumin–Cordycepin combination 150 µM
Co	Cordycepin
Co50	Cordycepin 50 µM
Co150	Cordycepin 150 µM
Cu	Curcumin
Cu50	Curcumin 50 µM
Cu150	Curcumin 150 µM
DMSO	Dimethyl sulfoxide
EGF	Epidermal Growth Factor
EGFR	Epidermal Growth Factor Receptor
EMT	Epithelial-to-Mesenchymal Transition
EpiCGS	Epithelial Cell Growth Supplement
FAK	Focal Adhesion Kinase
FGF-2	Fibroblast Growth Factor 2
GCO	Global Cancer Observatory
h	Hours
H_2_O_2_	Hydrogen Peroxide
HGEpiC	Human Gingival Epithelial Cells
HSC-4	Human oral squamous carcinoma cell line (HSC-4)
ITS	Insulin–Transferrin–Selenium
iNOS	Inducible Nitric Oxide Synthase
JNK	c-Jun N-terminal kinase
LDH	Lactate Dehydrogenase
MMP	Matrix Metalloproteinase
MTT	3-(4,5-Dimethylthiazol-2-yl)-2,5-diphenyltetrazolium bromide
NF-κB	Nuclear Factor kappa-light-chain-enhancer of activated B cells
NO	Nitric Oxide
OECM-1	Human oral squamous carcinoma cell line
OSCC	Oral Squamous Cell Carcinoma
PHI	Phase Holographic Imaging
ROS	Reactive Oxygen Species
RPMI	Roswell Park Memorial Institute medium
SD	Standard Deviation

## References

[1] Tan Y., Wang Z., Xu M., Li B., Huang Z., Qin S., Nice E.C., Tang J., Huang C. (2023). Oral squamous cell carcinomas: State of the field and emerging directions. Int. J. Oral Sci..

[2] Sun R., Dou W., Liu W., Li J., Han X., Li S., Wu X., Wang F., Xu X., Li J. (2023). Global, regional, and national burden of oral cancer and its attributable risk factors from 1990 to 2019. Cancer Med..

[3] George G.S., Patil A., Moirangthem R., Doibale P.N., Manjrekar A., Golapkar S.V., Panse N., Krishnatreya M., Mishra A., Singh A. (2025). Association of alcohol and different types of alcoholic beverages with the risk of buccal mucosa cancer in Indian men: A multicentre case-control study. BMJ Glob. Health.

[4] Ashraf S.A., Elkhalifa A.E.O., Siddiqui A.J., Patel M., Awadelkareem A.M., Snoussi M., Ashraf M.S., Adnan M., Hadi S. (2020). Cordycepin for health and wellbeing: A potent bioactive metabolite of *Cordyceps* medicinal fungus and its nutraceutical and therapeutic potential. Molecules.

[5] Binlateh T., Uppatcha N., Thepchai J., Pleungtuk Y., Noisa P., Hutamekalin P., Jitprasertwong P. (2022). Cordycepin attenuates migration and invasion of HSC-4 oral squamous carcinoma cells through autophagy-dependent FAK/Akt and MMP2/MMP9 suppression. J. Dent. Sci..

[6] Ho S.Y., Wu W.S., Lin L.C., Wu Y.H., Chiu H.W., Yeh Y.L., Huang B.M., Wang Y.J. (2019). Cordycepin enhances radiosensitivity in oral squamous carcinoma cells by inducing autophagy and apoptosis through cell cycle arrest. Int. J. Mol. Sci..

[7] Hewlings S.J., Kalman D.S. (2017). Curcumin: A review of its effects on human health. Foods.

[8] Ramos T.d.C.F., Dias R.B., Santos L.d.S., Valverde L.d.F., Nogueira R.L.R., Bastos I.N., Della Coletta R., Soares M.B.P., Souza B.S.d.F., dos Santos J.N. (2025). Curcumin triggers reactive oxygen species-mediated apoptosis and suppresses tumor growth in metastatic oral squamous cell carcinoma. Front. Oncol..

[9] Liu T., Long T., Li H. (2021). Curcumin suppresses the proliferation of oral squamous cell carcinoma through a specificity protein 1/nuclear factor-κB-dependent pathway. Exp. Ther. Med..

[10] Kim J.Y., Cho T.J., Woo B.H., Choi K.U., Lee C.H., Ryu M.H., Park H.R. (2012). Cu-induced autophagy contributes to the decreased survival of oral cancer cells. Arch. Oral Biol..

[11] Zhen L., Fan D., Yi X., Cao X., Chen D., Wang L. (2014). Curcumin inhibits oral squamous cell carcinoma proliferation and invasion via EGFR signaling pathways. Int. J. Clin. Exp. Pathol..

[12] Ni H., Hao R.L., Li X.F., Raikos V., Li H.H. (2018). Synergistic anticancer and antibacterial activities of cordycepin and selected natural bioactive compounds. Trop. J. Pharm. Res..

[13] Kaokaen P., Sorraksa N., Phonchai R., Chaicharoenaudomrung N., Kunhorm P., Noisa P. (2023). Enhancing neurological competence of nanoencapsulated *Cordyceps*/turmeric extracts in human neuroblastoma SH-SY5Y cells. Cell. Mol. Bioeng..

[14] Hussein N.I., Molina A.H., Sunga G.M., Amit M., Lei Y.L., Zhao X., Hartgerink J.D., Sikora A.G., Young S. (2024). Localized intratumoral delivery of immunomodulators for oral cancer and oral potentially malignant disorders. Oral. Oncol..

[15] Niţu C.D., Mernea M., Vlasceanu R.I., Voicu-Balasea B., Badea M.A., Raduly F.M., Rădiţoiu V., Rădiţoiu A., Avram S., Mihailescu D.F. (2024). Biomedical promise of sustainable microwave-engineered symmetric Curcumin derivatives. Pharmaceutics.

[16] Gabr S.A., Elsaed W.M., Eladl M.A., El-Sherbiny M., Ebrahim H.A., Asseri S.M., Eltahir Y.A.M., Elsherbiny N., Eldesoqui M. (2022). Curcumin modulates oxidative stress, fibrosis, and apoptosis in drug-resistant cancer cell lines. Life.

[17] Sato A., Shimotsuma A., Miyoshi T., Takahashi Y., Funayama N., Ogino Y., Hiramoto A., Wataya Y., Kim H.-S. (2023). Extracellular leakage protein patterns in two types of cancer cell death: Necrosis and apoptosis. ACS Omega.

[18] Connelly S.T., Macabeo-Ong M., Dekker N., Jordan R.C., Schmidt B.L. (2005). Increased nitric oxide levels and iNOS over-expression in oral squamous cell carcinoma. Oral Oncol..

[19] Zheng M., Ekmekcioglu S., Walch E.T., Tang C.H., Grimm E.A. (2004). Inhibition of nuclear factor-kappaB and nitric oxide by curcumin induces G2/M cell cycle arrest and apoptosis in human melanoma cells. Melanoma Res..

[20] Kim M.E., Lee J.S. (2025). Advances in the regulation of inflammatory mediators in nitric oxide synthase: Implications for disease modulation and therapeutic approaches. Int. J. Mol. Sci..

[21] Sendtner N., Seitz R., Brandl N., Müller M., Gülow K. (2025). Reactive Oxygen Species Across Death Pathways: Gatekeepers of Apoptosis, Ferroptosis, Pyroptosis, Paraptosis, and Beyond. Int. J. Mol. Sci..

[22] Duta C., Muscurel C., Dogaru C.B., Stoian I. (2024). Ferroptosis—A Shared Mechanism for Parkinson’s Disease and Type 2 Diabetes. Int. J. Mol. Sci..

[23] Chen C.W., Hsieh M.J., Ju P.C., Hsieh Y.H., Su C.W., Chen Y.L., Yang S.F., Lin C.W. (2022). Curcumin analog HO-3867 triggers apoptotic pathways through activating JNK1/2 signalling in human oral squamous cell carcinoma cells. J. Cell. Mol. Med..

[24] Utaipan T., Boonyanuphong P., Chuprajob T., Suksamrarn A., Chunglok W. (2020). A trienone analog of curcumin possesses ROS- and caspase-mediated apoptosis in human oral squamous cell carcinoma cells in vitro. Appl. Biol. Chem..

[25] Hsieh M.J., Ho H.Y., Lo Y.S., Lin C.C., Chuang Y.C., Abomughaid M.M., Hsieh M.C., Chen M.K. (2023). Semilicoisoflavone B induces apoptosis of oral cancer cells by inducing ROS production and downregulating MAPK and Ras/Raf/MEK signaling. Int. J. Mol. Sci..

[26] Du Y., Lai J., Su J., Li J., Li C., Zhu B., Li Y. (2024). Naringenin-induced oral cancer cell apoptosis via ROS-mediated Bid and Bcl-xl signaling pathway. Curr. Cancer Drug Targets.

[27] Tubtimsri S., Chuenbarn T., Manmuan S. (2025). Quercetin triggers cell apoptosis-associated ROS-mediated cell death and induces S and G2/M-phase cell cycle arrest in KON oral cancer cells. BMC Complement. Med. Ther..

[28] Tania M., Shawon J., Saif K., Kiefer R., Khorram M.S., Halim M.A., Khan M.A. (2019). Cordycepin downregulates Cdk-2 to interfere with cell cycle and increases apoptosis by generating ROS in cervical cancer cells: An in vitro and in silico study. Curr. Cancer Drug Targets.

[29] Kim S.O., Cha H.J., Park C., Lee H., Hong S.H., Jeong S.J., Park S.H., Kim G.Y., Leem S.H., Jin C.Y. (2019). Cordycepin induces apoptosis in human bladder cancer T24 cells through ROS-dependent inhibition of the PI3K/Akt signaling pathway. Biosci. Trends.

[30] Dong J., Li Y., Xiao H., Luo D., Zhang S., Zhu C., Jiang M., Cui M., Lu L., Fan S. (2019). Cordycepin sensitizes breast cancer cells toward irradiation through elevating ROS production involving Nrf2. Toxicol. Appl. Pharmacol..

[31] Tung K.L., Wu S.Z., Yang C.C., Chang H.Y., Chang C.S., Wang Y.H., Huang B.M., Lan Y.Y. (2022). Cordycepin induces apoptosis through JNK-mediated caspase activation in human OEC-M1 oral cancer cells. Evid.-Based Complement. Altern. Med..

[32] Zhang Z., Li K., Zheng Z., Liu Y. (2022). Cordycepin inhibits colon cancer proliferation by suppressing MYC expression. BMC Pharmacol. Toxicol..

[33] Banerjee M., Singh P., Panda D. (2010). Curcumin suppresses the dynamic instability of microtubules, activates the mitotic checkpoint and induces apoptosis in MCF-7 cells. FEBS J..

[34] Gupta K.K., Bharne S.S., Rathinasamy K., Naik N.R., Panda D. (2006). Dietary antioxidant curcumin inhibits microtubule assembly through tubulin binding. FEBS J..

[35] Jackson S.J., Murphy L.L., Venema R.C., Singletary K.W., Young A.J. (2013). Curcumin binds tubulin, induces mitotic catastrophe, and impedes normal endothelial cell proliferation. Food Chem. Toxicol..

[36] Chen Q., Lu G., Wang Y., Xu Y., Zheng Y., Yan L., Jiang Z., Yang L., Zhan J., Wu Y. (2009). Cytoskeleton disorganization during apoptosis induced by Cu in A549 lung adenocarcinoma cells. Planta Medica.

[37] Ridho F.M., Syachputra A.J., Fahrudin P., Nurhuda A., Nurliana N., Latuamury N.S. (2024). In vitro and in vivo effects of curcumin on oral cancer: A systematic review. Curr. Biomed..

[38] Tsang R.K., Tang W.W., Gao W., Ho W.K., Chan J.Y., Wei W.I., Wong T.S. (2012). Curcumin inhibits tongue carcinoma cell migration and invasion through downregulation of matrix metallopeptidase 10. Cancer Investig..

[39] Lee A.Y., Fan C.C., Chen Y.A., Cheng C.W., Sung Y.J., Hsu C.P., Kao T.Y. (2015). Curcumin inhibits invasiveness and epithelial–mesenchymal transition in oral squamous cell carcinoma by reducing matrix metalloproteinase-2 and -9 and modulating the p53–E-cadherin pathway. Integr. Cancer Ther..

[40] Ohnishi Y., Sakamoto T., Zhengguang L., Yasui H., Hamada H., Kubo H., Nakajima M. (2020). Curcumin inhibits epithelial–mesenchymal transition in oral cancer cells via c-Met blockade. Oncol. Lett..

[41] Dudás J., Fullár A., Romani A., Pritz C., Kovalszky I., Schartinger V.H., Sprinzl G.M., Riechelmann H. (2013). Curcumin targets fibroblast–tumor cell interactions in oral squamous cell carcinoma. Exp. Cell Res..

[42] Enasescu D.S., Voicu-Balasea B., Rus-Hrincu F., Radulescu R., Popa A., Moisa M.R., Coculescu B.-I., Imre M.M., Pituru S.M., Ripszky A. (2024). Exploring Comparatively the Cytotoxicity of Milled and 3D-Printed PMMA-Based Resins to Oral Squamous Cancer Cells—What Signals Could the AKT/mTOR Pathway Send Us through Its Downstream Effector GSK-3?. Rom. J. Oral Rehabil..

[43] AAT Bioquest, Inc. Quest Graph™ IC50 Calculator. https://www.aatbio.com/tools/ic50-calculator.

